# Arbuscular Mycorrhizal Fungi Mediate Drought Tolerance and Recovery in Two Contrasting Carob (*Ceratonia siliqua* L.) Ecotypes by Regulating Stomatal, Water Relations, and (In)Organic Adjustments

**DOI:** 10.3390/plants9010080

**Published:** 2020-01-08

**Authors:** Abderrahim Boutasknit, Marouane Baslam, Mohamed Ait-El-Mokhtar, Mohamed Anli, Raja Ben-Laouane, Allal Douira, Cherkaoui El Modafar, Toshiaki Mitsui, Said Wahbi, Abdelilah Meddich

**Affiliations:** 1 Laboratory of Biotechnology and Plant Physiology, Faculty of Sciences Semlalia, Cadi Ayyad University, BP: 2390, Marrakesh 40000, Morocco; 2 Department of Applied Biological Chemistry, Faculty of Agriculture, Niigata University, Niigata 950-2181, Japan; 3 Department of Life and Food Sciences, Graduate School of Science and Technology, Niigata University, Niigata 950-2181, Japan; 4 Laboratory of Botany and Plant Protection, Faculty of Science, BP. 133, Ibn Tofail University, Kenitra 14000, Morocco; 5 Laboratory of Biotechnology and Molecular Bioengineering, Faculty of Sciences and Techniques, Cadi Ayyad University, BP: 2390, Marrakesh 40000, Morocco

**Keywords:** native mycorrhiza, chlorophyll fluorescence, indigenous species adaptability, antioxidative systems, drought recovery, tolerance, water limitation

## Abstract

Irregular precipitation and drought caused an increase in tree mortality rates in multiple forest biomes with alterations in both ecosystem services and carbon balance. Carob (*Ceratonia siliqua*) growth and production in arid and semi-arid ecosystems are likely affected by climate change-induced droughts. Understanding the physiological responses of drought-induced early-stage tree death and strategies to enhance drought tolerance and optimize growth will help tree improvement programs. Mycorrhizal inoculation has a pronounced impact on plant growth, water absorption, mineral nutrition, and protection from abiotic stresses. However, a better understanding of these complex interconnected cellular processes and arbuscular mycorrhizal fungi (AMF)-mediated mechanisms regulating drought tolerance in plants will enhance its potential application as an efficient approach for bio-amelioration of stresses. The objectives of this work were to elucidate the different effects of autochthone AMF on inorganic solute and water content uptakes, organic adjustments (sugar and proteins content), leaf gas exchange (stomatal conductance and efficiency of photosystems I and II), and oxidative damage of two contrasting ecotypes of carob seedlings: coastal (southern ecotype (SE)) and in-land (northern ecotype (NE)) under control (C), drought (by cessation of irrigation for 15 days (15D)), and recovery (R) conditions. Our findings showed that AMF promoted growth, nutrient content, and physiological and biochemical parameters in plants of both ecotypes during C, 15D, and R conditions. After four days of recovery, stomatal conductance (g_s_), the maximum photochemical efficiency of PSII (F_v_/F_m_), water content, and plant uptake of mineral nutrients (P, K, Na, and Ca) were significantly higher in shoots of mycorrhizal (AM) than non-mycorrhizal (NM) control plants. Consequently, AMF reduced to a greater degree the accumulation of hydrogen peroxide (H_2_O_2_) and oxidative damage to lipid (malondialdehyde (MDA)) content in AM than NM plants in NE and SE, after recovery. Altogether, our findings suggest that AMF can play a role in drought resistance of carob trees at an early stage by increasing the inorganic solutes (P, K, Na, and Ca), water content uptake, organic solutes (soluble sugars and protein content), stomatal conductance, and defense response against oxidative damage during re-watering after drought stress.

## 1. Introduction

Currently, drought is one of the most frequent and severe abiotic stresses negatively affecting many physiological and biochemical parameters, plant growth, and development in arid and semi-arid Mediterranean ecosystems [1,2]. In addition, these ecosystems are subject to particular climatic conditions and anthropogenic pressure, leading to a decline in forest cover and irreversibly to serious environmental consequences such as rapid soil erosion and desertification [3]. Drought in these regions is becoming extreme, longer, and more frequent. This alters the biodiversity of vegetation, and often makes the reforestation with original species difficult [2]. Faced with this critical situation, reforestation is more than ever a major challenge and an absolute necessity. The use of pioneering tree species, adapted to climatic hazards and able to settle on eroded soils, remains the most recommended solution. The carob (*Ceratonia siliqua*) is considered an important component of vegetation for economic and environmental reasons [4]. Carob products recently became valued as a human food, as they are an excellent source of bioactive compounds and also a cocoa substitute since they do not contain caffeine and theobromine. Also, carob can be used as a livestock feed in agroforestry systems. This tree was widely known as the “black gold” of several Mediterranean regions owing to its beneficial by-products for humans [5]. Currently, carob is included in the European Union (EU) and Australia aid programs. The tree shows some outstanding features as one of the high-potential species in reforestation actions to enhance and replace drought-sensitive species and in the rehabilitation of marginal areas of the Mediterranean basin [6,7]. However, unfortunately, this plant is not commonly used in the reforestation programs of many countries where other reforestation programs (i.e., species adaptability, soil grading, fertilizing, revegetating) failed [8]. Furthermore, studies characterizing carob cultivars are scarce worldwide. Hence, the selection and use of endemic species could improve the success rate of these environmental actions [6]. Carob is traditionally beneficial to the agricultural economy since it is a drought- and temperature-tolerant plant. The drought tolerance of carobs is based on morphological, physiological, and biochemical mechanisms to prevent stress. Furthermore, carobs are able to develop other strategies to overcome water constraints such as their association with mycorrhizal fungi [9,10].

Agricultural sustainability and the management of natural resources are garnering increasing support by integrating environmental health, economic profitability, and social equity. By designing biologically integrated agroecosystems that rely more on the soil cycling of nutrients, it would be possible to maintain an economical production system. Arbuscular mycorrhizal fungi (AMF) are widely used as biofertilizers to increase plant yield and strengthen crop resistance to adverse environmental conditions in agricultural and forestry programs [11,12,13], and they represent an under-exploited potentially useful way to boost global food security. Studies on plant–AMF associations showed that over 70% of vascular plant species can establish a symbiotic association that is mutually beneficial to both host plant and fungus, especially under water stress conditions [14,15]. AMF increase plant growth and could confer drought resistance [16,17], owing to the well-developed extraradical mycorrhizal mycelia that proliferate in the bulk soil beyond the rhizosphere, thus forming a network specialized in the acquisition of water and mineral nutrients from soil [18]. The mycorrhizal symbiosis improves plant performance under drought through increasing stomatal conductance and photosynthesis of host plants [19] and through enhancing plant tissue protection against oxidative damages [20]. Mycorrhizal plants regulate stomatal aperture under drought to avoid excessive water loss and wilting that limits CO_2_ influx [21]. Moreover, AM-mediated drought responses are likely linked to phosphorus content, coordination between root hydraulic conductivity and shoot stomatal conductance (g_s_), aquaporin expression, and/or signaling molecules [22,23,24]. Wu and Zou [25] indicated that AMF could help the host plant to enhance drought tolerance via biochemical mechanisms including osmotic adjustment and antioxidant systems. Under drought conditions, plants alter water relations by synthesizing compatible solutes (e.g., sugar, proline, amino acid) to sustain turgor pressure and cellular functions for maintenance of metabolic functions [26,27]. This recovery of metabolic activities is facilitated by the osmotic adjustment after stress relief [27]. Studies focusing on the impact of metabolic alterations, antioxidative processes, and osmolyte dynamics in water-stressed plants and re-watering are limited. Furthermore, the influence of AMF on plant drought resistance in tree species with different photosynthetic capabilities, physiological mechanisms, and antioxidant defense systems remains unclear, and the effects of AMF on carob seedlings are yet to be studied under drought and recovery conditions. Therefore, the goal of the present study was to evaluate the effect of extreme drought and rehydration (recovery) of two mycorrhizal and non-mycorrhizal carob ecotypes at the physiological, mineral nutrition, and biochemical levels, and at the cellular level by characterizing the components of oxidative stress. Deciphering the AMF-mediated mechanisms in the plant protection responses and metabolic pathways under unfavorable conditions is required to gain insight into their potential, and it will open up new approaches to exploit AMF as a bioprotective tool against drought in sustainable global food security.

## 2. Results

### 2.1. Mycorrhizal Status and Plant Growth Performance

The frequency (F) and intensity (I) of the root seedlings inoculated with AMF showed no significant differences among the mycorrhizal–carob seedlings in control (C), drought by cessation of irrigation for 15 days (15D), and recovery (R) conditions in both ecotypes (Table 1). The frequency (>60%) and intensity (>32%) of mycorrhization remained high and were not affected in both ecotypes even at the end of 15D and after recovery conditions. Non-mycorrhizal (NM) plants showed an absence of mycorrhizae in both ecotypes under C, 15D, and R conditions. For both ecotypes, the frequency of AM colonization was 62% under C, and ca. 62–65% under 15D. Similarly, there were no differences in the intensity of mycorrhizal colonization among all the conditions of the study.

Under normal conditions (C), AMF did not affect above-ground biomass (shoot dry weight (SDW)) in the northern ecotype (NE). Significant AMF effects on the above- and below-ground biomasses were observed for both ecotypes under drought and recovery conditions. Under the 15D treatment, shoot (SDW) and root (RDW) dry matter remained stable in NM plants, while AMF increased mean plant root and shoot biomasses in carob plants (*p* < 0.05) (Table 1). Indeed, after the 15D condition, the percentages of shoot dry weight (SDW) increment variation were lower in NM than AM plants by 31% in NE and 24% in southern ecotype (SE). Root biomass accumulation was improved by AMF inoculation under water stress with 27% and 25% dry weight losses in NE and SE, respectively, compared to NM plants. In both ecotypes, the inoculation of carob seedlings with AMF significantly (*p* < 0.05) alleviated the deleterious effects of water stress on growth and biomass accumulation independently of the ecotype. For both C and 15D treatments, there was a significant increase (*p* < 0.05) of root length (RL) in AM and NM plants in the SE ecotype as compared with NE. Such an increase was more pronounced in mycorrhizal plants (*p* < 0.05). The application of AMF in prolonged water stress and recovery conditions caused significant differences (*p* < 0.05) in RL between SE and NE plants, with the latter growing 16% more than the former.

### 2.2. AM and NM Plant Mineral Absorption Efficiency under Drought and Recovery Regimes

To evaluate the nutritional status of AM and NM counterparts, we analyzed the mycorrhizal impact on leaf P, K, Ca, and Na accumulation (Table 2) under normal, drought imposition, and re-watering conditions. The nutrient concentrations in leaves of carob tree seedlings significantly varied depending on the water regime (*p* < 0.05) and mycorrhizal status (*p* < 0.05) in both ecotypes (Table 2). Under maintained water stress, carob plants significantly increased P, K, and Ca levels as compared to plants grown under the control condition. The presence of AM induced a higher accumulation of all the studied nutrients compared to NM plants regardless of the water regime. As a result, we found that the application of drought in AM plants led to a significant 1.8-fold increase in P, K, and Na and a 1.3-fold increase in Ca levels as compared to NM plants in both ecotypes. In addition, P, K, Na, and Ca content in AM vs. NM plants after 15D increased ((9% vs. 4%), (40% vs. 24%), (12% vs. 11%) and (8% vs. 6%) in NE and (9% vs. 8%), (39% vs. 29%), (11% in both AM and NM), and (15% vs. 8%) in SE) compared to those under the C treatment (Table 2). Under water stress, the association of NE with AMF had significantly higher leaf Na and Ca content relative to the other SE ecotype, whereas there were no significant differences in leaf P and K nutrient levels between both ecotypes. It should be noted that no obvious differences in nutrient content were recorded at the end of the recovery condition compared with 15D treatment independently of the cultivar and mycorrhizal status. Mycorrhizal association under the recovery condition increased P, K, Na, and Ca content by 1.5%, 3.5%, 1.4%, and 5.6% in NE and 3.2%, 0.8%, 0.1%, and 2.5% in SE, respectively, relative to mycorrhizal plants under the 15D condition.

### 2.3. Leaf Water Potential and Relative Water Content

**Figure 1:**
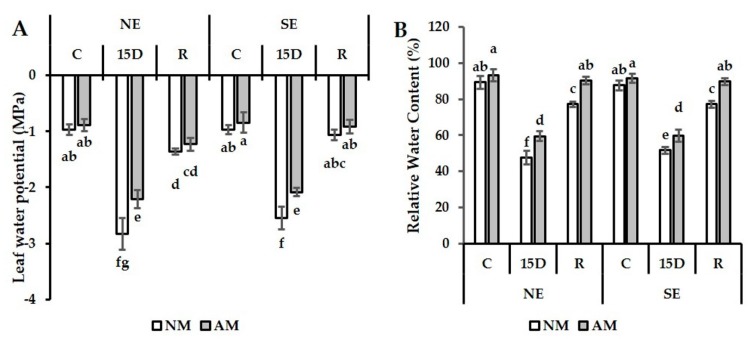
(**A**) Leaf water potential (Ψ_Leaf_) and (**B**) relative water content (RWC) in non-mycorrhizal (NM) and arbuscular mycorrhizal (AM) carob ecotypes (northern and southern (NE and SE)) grown under control (C), 15 days of drought (15D), and recovery (R) conditions. Values with different letters indicate significant differences between treatments at *p* ≤ 0.05 by the Tukey test. Data represent the means ± standard error (SE) (*n* = 5).

The leaf water potential (Ψ_Leaf_) was ca. −1, and relative water content (RWC) was approximately 93% under the well-watered condition, and neither parameter was markedly affected by AMF colonization (Figure 1A,B). Under 15D, drought stress significantly reduced Ψ_leaf_ and RWC in AM and NM carob plants (*p* < 0.05) in both ecotypes, but such a reduction was more pronounced in the absence of AMF. Indeed, Ψ_leaf_ was dramatically decreased in NM by ca. 30% in NE and ca. 15% in SE ecotypes as compared to AM plants (Figure 1A). Under the recovery phase, we observed that AM and NM plants of the SE ecotype showed a much greater capacity to return to the initial state than NE. The application of the mycorrhizal inoculum allowed recovery of 91% in SE and 60% in NE compared to the initial state. Drought stress markedly decreased leaf RWC, and this was observed to a greater extent (ca. 47% in NE and 41% in SE non-mycorrhizal ecotypes as compared to ca. 35% in both NE and SE ecotypes treated with AMF) (Figure 1B). Under 15D, AM plants exhibited an enhancement of RWC by 25% in NE and 16% in the SE ecotypes when compared to NM plants (Figure 1B). Under prolonged water stress, our data showed that non-mycorrhizal SE maintained slightly higher RWC (ca. 9%) than non-mycorrhizal NE (Figure 1B). Under the recovery condition, RWC was significantly increased at a higher level in AM than NM plants. This increase was ca. 17% in both NE and SE. As a result, the alterations in water parameters were more drastic in the stressed non-mycorrhizal plants than in the stressed mycorrhizal plants. Furthermore, our data showed that the SE ecotype had a better response to drought stress and recovery conditions than NE.

### 2.4. AM-Mediated Stomatal Conductance and Efficiency of Photosystems I and II in Coping with Drought Stress

**Figure 2:**
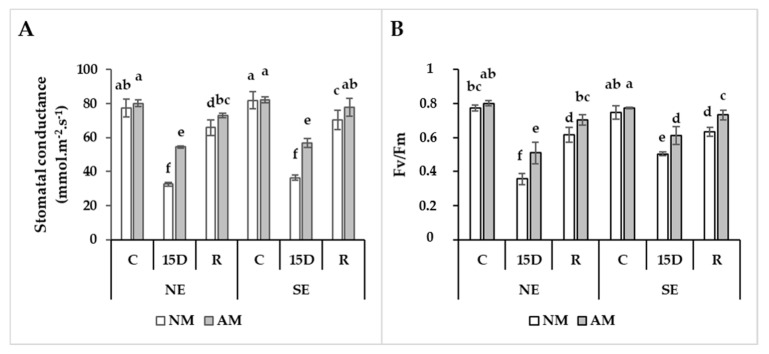
Effect of drought stress (15D) and rehydration (R) on stomatal conductance (**A**) and chlorophyll fluorescence (F_v_/F_m_) (**B**) in non-mycorrhizal (NM) and arbuscular mycorrhizal (AM) carob ecotypes (NE and SE). Data (means ± SE, *n* = 5) followed by different letters above the bars indicate significant differences between treatments at *p* ≤ 0.05 by the Tukey test.

The results of the physiological traits related to photosynthesis showed no significant variations in stomatal conductance (g_s_) and quantum efficiency of photosystem II (F_v_/F_m_) between AM and NM plants under control conditions (Figure 2A,B). However, drought stress induced a severe decline in both physiological traits g_s_ and F_v_/F_m_ in both NE and SE ecotypes. The magnitude of decline was greater for NM (58% in NE vs. 56% in SE) than AM (31% in SE vs. 32% in NE) plants. Under the 15D treatment, AM-treated plants enhanced the g_s_ by ca. 60% in NE and 57% in SE as compared to NM plants. Under both conditions C and 15D, the SE ecotype exhibited higher g_s_ values than NE ecotypes regardless of the presence or absence of AM. By the end of recovery condition, the g_s_ in AM plants showed a more rapid ability (ca. 95% in SE and 90% in NE) to return to the initial state than NM plants (ca. 80% in both SE and NE).

In both ecotypes, 15 days of water restriction decreased F_v_/F_m_ to a greater degree in NM plants (117% in NE vs. 49% in SE) (Figure 2B) than AM plants. Compared to NM, mycorrhizal colonization allowed an increase of F_v_/F_m_ by ca. 22% in 15D treatments (Figure 2B). After four days of recovery, in both ecotypes, AM plants showed a greater ability to return F_v_/F_m_ to the control values (ca. 86% in NE vs. 90% in SE of recovery) than NM plants. As a result, during drought stress, the physiological parameters (g_s_ and F_v_/F_m_) decreased more in the NE than in the SE ecotype. Mycorrhizal plants in both ecotypes showed higher values when stress was imposed and a greater capacity for recovery (at the same levels as C values with or without AMF) after a previous drought period than NM.

### 2.5. Total Soluble Sugar and Protein Contents

**Figure 3:**
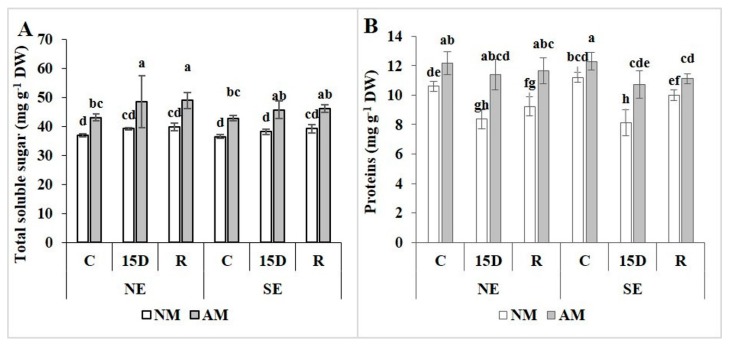
Effect of drought stress (15D) and rehydration (R) on total soluble sugars (**A**) and protein content (**B**) in non-mycorrhizal (NM) and arbuscular mycorrhizal (AM) carob ecotypes (NE and SE). Data (means ± SE, *n* = 5) followed by different letters above the bars indicate significant differences between treatments at *p* ≤ 0.05 by the Tukey test.

Leaf carbohydrate analysis in AM carob ecotypes during drought stress and the recovery period revealed that the association of plants with the AMF always increased the content of total soluble sugar independently of the treatments (C, 15D, and R) (Figure 3A). This accumulation reached higher values in AM plants under 15D (ca. 25% in NE and 20% in SE) than NM plants. However, for both ecotypes, there were no differences in leaf soluble sugars in non-mycorrhizal plants regardless of water status. Protein content in non-mycorrhizal plant leaves was significantly reduced during stress imposition (a reduction of 40% in SE and 21% in NE) and recovery (ca. 13%) (Figure 3B). However, the mycorrhizal contribution allowed protein content to be maintained under drought stress and recovery at the relatively same level as controls (not significant (NS); *p* > 0.05) (Figure 3B). Both ecotypes showed the same patterns in response to the presence/absence of AM and drought/recovery treatments.

### 2.6. Hydrogen Peroxide and Malondialdehyde Concentrations

**Figure 4:**
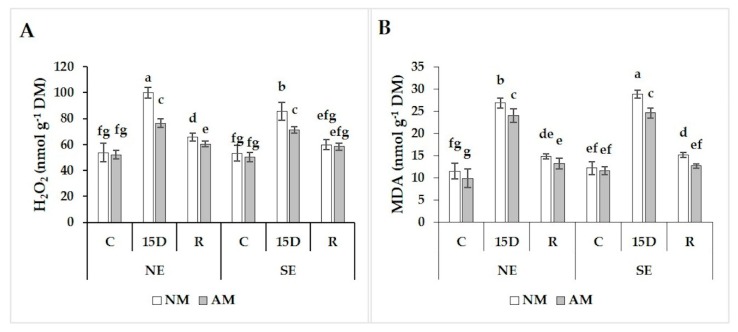
Effect of drought stress (15D) and rehydration (R) on hydrogen peroxide (H_2_O_2_) (**A**) and malondialdehyde (MDA) (**B**) concentrations in non-mycorrhizal (NM) and arbuscular mycorrhizal (AM) carob ecotypes (NE and SE). Data (means ± SE, *n* = 5) followed by different letters above the bars indicate significant differences between treatments at *p* ≤ 0.05 by the Tukey test.

Accumulation of hydrogen peroxide (H_2_O_2_) and malondialdehyde (MDA) is an indicator of oxidative stress in plant tissues related to the formation of reactive oxygen species. Under the control condition, there was no difference between AM and NM carob plants for H_2_O_2_ and MDA (Figure 4A,B) concentrations. Nonetheless, 15D caused significantly (*p* < 0.05) higher H_2_O_2_ and MDA accumulation in carob seedling leaves. These increases were significantly greater (*p* < 0.05) in NM plants than AM plants in both ecotypes (Figure 4A,B). Indeed, NM plants had higher H_2_O_2_ levels (31% in NE vs. 20% in SE) compared to AM plants. Similarly, we observed a significant enhancement in MDA accumulation in NM plants by 12% in NE and 17% in SE as compared to AM plants (Figure 4B). The increases in H_2_O_2_ and MDA following exposure to drought were largely reversed under recovery conditions. In both ecotypes, H_2_O_2_ and MDA concentrations decreased to lower levels in AM than NM plants to reach close to control values. Under recovery, AM plants significantly decreased H_2_O_2_ (8% in NE vs. 2% in SE) and MDA (13% in NE vs. 20% in SE) content as compared to NM plants. Furthermore, the AM and NM plants of the SE ecotype showed a better ability to tolerate drought damages than NE ecotype.

## 3. Discussion

Environmental constraints affect plant growth and development, which consequently hamper their productivity. Exposure of plants to water stress is considered the single most devastating environmental stress, reducing their productivity more than any other environmental stress [28]. In the field, most trees lack root hairs and are strongly dependent on AM symbiosis. Furthermore, carob is considered a species resistant to water limitation owing to its many adaptative mechanisms such as the optimization of water-spender strategies [29,30], thus influencing the morphology, physiology, and biochemical processes of the plant [30,31]. AMF are important soil microorganisms in natural ecosystems, by forming symbiotic associations with most terrestrial vascular plants. The underground hyphal networks formed by AMF can influence plant growth, nutrient acquisition, and plant–plant interactions. Currently, several studies reported that AM symbiosis protects host plants against detrimental effects of water deficit. This is mainly due to the combination of nutritional, physiological, and biochemical alterations [32,33]. Nonetheless, the recovery process after prolonged water stress is poorly studied, and the role played by AMF in this process is almost unknown. Here, we report on the impact of AMF autochthons on inorganic and water content uptake, leaf gas exchange, and oxidative damage of two contrasting ecotypes of carob seedlings.

In this report, no mycorrhizal structure was observed in the roots of non-AM carob trees, but the inoculated plants were successfully infected by the native mycorrhizal consortium quickly reaching 60% in both ecotypes. The entry points and vesicles did not significantly increase during the prolonged water stress and recovery condition probably owing to their short-term (up to 15 and four days of drought and recovery, respectively), implying that low duration of drought does not appear to favor or discourage colonization [34]. This result is in line with the finding of Quiroga et al. [35], who reported that water stress had no effect on root colonization during 12 days of inoculation. Additionally, Augé [34] revealed that long-term watering cessation reduced the root colonization by AMF. Wu and Xia [36] also observed a reduction of *Glomus versiforme* colonization after drought conditions following full irrigation. Our results are also in accordance with other finding using different AM and plants species such *G. mosseae* (actually *Funneliformis mosseae* (Nicol. and Gerd.)) and *G. intraradices* (actually *Rhizophagus intraradices* (Schenck and Smith) Walker and Schüβler comb. nov.) with *Casuarina glauca* [37], *G. clarum* with tomato [38], *G. etunicatum* with pistachio [39], and *F. mosseae*, *R. fasciculatus*, and *R. intraradices* with carob [10] under drought stress. The phenomenon could be attributed to the unchanged carbon availability from host plants. In contrast, other studies showed a negative effect of prolonged water stress on AM development [15,40,41,42,43]. A potential explanation to the limited negative responses of AMF to drought can be related to the length of drought, the mycorrhiza inocula/accessions used in our study having “pre-adaption” properties to water shortage, and/or the higher initial carbon investment of plants into the development of mycorrhizal structures. All in all, the efficiency of the AMF is often measured in terms of host plant growth and biomass improvement under water-deficit stress [35].

In the present investigation, the application of water stress more negatively affected growth (SH, RL, SDW, and RDW) in NM plants than AM plants in both ecotypes. AMF boosted plant growth and the accumulation of above- and below-ground biomasses, which is consistent with the results of previous studies showing that AMF can alleviate the negative effects of drought and improve plant growth [39,40,44,45,46,47,48]. These results could be attributed to drought-associated improvement in the leaf carbon assimilation rate. The boosting of plant growth by AMF could be due to changes in both the photosynthesis and the antioxidant capacities of plants. Moreover, Lui et al. [47] reported that AM plants grown under well-watered and water stress conditions enhanced growth after seven days of drought and recovery for 30 days, and this was attributable to the improvement of phosphorus nutrition [49] and water uptake owing to the efficiency of extra-radical mycelial to extract water from soil even under water deficiency conditions [15,43]. In fact, the most obvious benefit from extraradical hyphae is that they can extend beyond depletion zones to absorb and transport the nutrient elements to intracellular arbuscules in colonized cortical cells [39]. Indeed, our finding showed that AMF significantly enhanced g_s_ and F_v_/F_m_, which could, in turn, enhance leaf carbon assimilation rate. This result is consistent with C3 crops under both well-watered and water stress conditions [15]. The recovery condition did not significantly influence the biomass in either ecotype. This might be because growth and biomass accumulation are long-term processes; thus, the short-term recovery condition may not greatly influence growth and biomass production. In terms of inter-ecotype drought susceptibility, NM and AM SE ecotype showed a better adaptation to drought stress and recovery period than the NE ecotype. Indeed, the SE ecotype presented higher RL than NE under drought and recovery conditions. Kozlowski and Pallardy [50] and Hartmann [51] demonstrated that tree species that better adapted to dry climatic regimes generally have higher root system attributes (e.g., root volume, root biomass, capacity to produce adventitious roots, root structure, rooting depth) than species that are more suited to mesic climatic conditions. Furthermore, Olmo et al. [52] showed a positive correlation between tree and shrub species’ fine roots and biomass increase under drought conditions. Our data suggest that carob growth under drought and recovery conditions is dependent on water status and ecotype interaction.

A prominent role of the symbiotic relationship is to transfer nutrients, and AMF colonization is widely considered to stimulate nutrient uptake in plants. Our results revealed that AMF showed the capability to boost the uptake of inorganic macro- and micro-nutrients under drought and recovery, specifically that of phosphate. The positive role of AMF on the net accumulation of nutrients in P was 10% in both NE and SE, while K was 40% in both ecotypes, and it was 21% in NE vs. 11% in SE for Na in leaves of AM plants. Studies reported that mycorrhizal symbiosis positively increased the concentrations of nutrients in several species under drought stress [53,54,55,56,57]. During the recovery condition, the nutrient uptake in the NE ecotype was quick and similar to that recorded under water stress compared to SE plants. Mycorrhizal contribution to nutrient uptake (P and K) was higher in NE than SE under water stress and after water resupply (recovery), explaining the better growth performance and biomass accumulation of mycorrhizal NE under water stress and recovery conditions. Le Pioufle et al. [49] observed that improved P nutrition of plants due to mycorrhizal symbiosis can help plants to improve water uptake and to mitigate the impact of drought stress and maintain growth. Neumann and George [56,58] showed that nutrient deficiency under prolonged water stress was ameliorated by AMF inoculation and, after water resupply, the AM plants showed a faster recovery. Similarly, in this study, we observed that plants inoculated with AMF and subjected to prolonged water stress and recovery had improved content of P, K, Na, and Ca in both carob ecotype leaves. Similar studies showed that growth improvement by AM can be related to increasing the essential nutrients and water absorption [18,59]. In addition, El-Mesbahi et al. [60] reported that improved K absorption in mycorrhizal plants could be a mechanism for water transport by mycorrhizal hyphae, because the supply of extra K always increased root hydraulic conductivity more in AM plants than NM plants, regardless of the water regime.

Our results showed that the water uptake (potential and the relative water content) was more largely increased in AM plants than NM plants under water stress conditions, while, under well-watered conditions, no significant differences were observed. It should be noted that, during the recovery condition, the AM carob ecotypes maintained higher Ψ_Leaf_ and RWC to remedy the injury that was caused by the water stress condition. Previous studies demonstrated that AM plants have higher levels of water status than NM under dried soils, more than under the saturated soil conditions [15,43,47,61]. Our results were in agreement with Liu et al. [47] suggesting that AMF could improve the absorption and the transportation of water under water stress and recovery conditions. The AMF symbiosis leads to higher root hydraulic conductivity, which is explained by the effective absorption of soil water and transport to the plant [34]. This extra absorption results from a larger root system due to AMF hyphae, which can increase the exploration area beyond the root zone, thus increasing the available volume of the soil solution [15,18,62]. AMF hyphae may compensate for the inhibition of aquaporin activity in AM plants under a water deficit [63]. Augé [34] and Zhu et al. [61] reported that the higher levels of RWC in AM plants may be beneficial for moving water to the evaporating surfaces and further opening the stomata. On the other hand, it is known that dehydration avoidance under drought stress is a consequence of a tight balance between stomatal movements, root water uptake capacity, and water distribution throughout plant tissues [19,61,64]. Previous studies found that AM plants often show higher stomatal conductance compared to NM plants under water stress [18,62].

Here, we showed that, during drought stress conditions, stomatal conductance was significantly reduced in stressed plants. The AM contributed to significantly greater increases in g_s_ than NM plants under stress and rapid recovery to the control levels in both ecotypes. A meta-analysis of 460 studies carried out by Augé et al. [62] showed that stomatal conductance data for amply watered or drought conditions revealed an increase of 24% in stomatal conductance of AM plants as compared to NM plants. The increase in stomatal conductance of AM plants, corroborated in this study, is attributed to the higher leaf phosphorus (P) concentrations [62]. Meddich et al. [18] demonstrated that the higher stomatal conductance of AM plants is associated with higher Ψ_Leaf_ and/or higher RWC, as well as greater osmotic adjustment. In this respect, it is worth noting that AMF influence the plant hormonal balance involved in the regulation of stomatal functions under water stress conditions [65,66,67]. Ouledali et al. [68] reported that ABA levels were lower in AM plants than in NM plants during unstressed, severe, and recovery conditions, which could lead to increases in transpiration and root water absorption.

In line with other studies, the higher water content and stomata opening in AM plants led to higher efficiency of photosystem II (PSII) (F_v_/F_m_) under water stress [62,69] and recovery [47] conditions. We found that AM fungus used in this study increased F_v_/F_m_ in plants exposed to long water stress conditions. Importantly, the F_v_/F_m_ in AM plants increased more rapidly under recovery conditions than NM plants. This result demonstrates that AM plants maintained a normal utilization of light energy in photochemical processes under severe water stress and recovery. Some studies demonstrated that, under prolonged water stress conditions, AMF inoculation increased the maximum quantum yield of PSII [40,48,70]. It was established that AMF inoculation can enhance drought tolerance in AM plants by ameliorating to some degree the injury caused to the photosystem reaction centers [71]. Indeed, the rapid improvement of F_v_/F_m_ in AM plants observed during recovery conditions was most likely attributed to attenuate cellular damage [72] and by protecting PSII [73].

AMF increased the water and nutrition content available to their host plants, as well as total soluble sugars (osmotic adjustment), under water stress conditions in order to cope and recover from drought stress conditions [74]. In the present study, we observed that AM carob plants accumulated more total soluble sugars during the prolonged water stress and recovery conditions, likely to maintain high tissue hydration [10] and to increase the photosynthetic activity [35,53,75]. Hexoses function as osmoprotectants and signals to induce plant stress responses and reduce reactive oxygen species (ROS) damage [76]. They contribute to the maintenance of turgor pressure, required for cell expansion and the synthesis of cell-wall components, including cellulose [76]. It was reported that total soluble sugar content regulated by AM plants plays a critical role in maintaining high leaf osmotic potential [48]. The regulation of the accumulation of soluble sugar content in AM plants is a mechanism deployed by carob seedlings in order to maintain high Ψ_Leaf_ in soils with low water potentials [10,29,30]. Our finding suggests that soluble sugar content in carob leaf tissues was mediated by AMF inoculation before being subjected to water deficit stress (during the control conditions). Moreover, our results showed a higher content of soluble proteins in the leaves of AM plants than NM plants under water stress and recovery conditions in both ecotypes. This difference in increase of protein levels could, at least partially, explain the ability of AMF to enhance the non-enzymatic antioxidant defense system [77,78]. Drought stress impedes plant growth through peroxidative damage [79]; nonetheless, AMF can reduce oxidative stress by increasing antioxidant enzyme activities in host plants [39,80,81]. MDA levels could indicate the magnitude of a plant’s exposure to peroxidative damage caused by drought stress [82]. In the present work, H_2_O_2_ and MDA were highly accumulated in NM plants compared to under water stress and recovery conditions in both ecotypes. In contrast, AMF colonization decreased H_2_O_2_ and MDA content after the recovery period, reaching NM control levels. Several studies indicated that the low H_2_O_2_ and MDA accumulation observed in AM plants was correlated with water stress tolerance [39,81,83]. Reduced H_2_O_2_ and MDA content in AM plants might be due to better water availability and osmoregulation and higher antioxidative defense systems [43,84,85]. In the present study, higher leaf water, ion homeostasis, and osmotic organic content in AM plants were associated with lower accumulation of H_2_O_2_ and MDA, indicating lower oxidative damage in the colonized plants. Notably, AM plants showed a rapid recovery to low levels of H_2_O_2_ and MDA after the drought stress period, allowing drought tolerance in inoculated carob seedlings.

## 4. Material and Methods

### 4.1. Plant Material and Experimental Design

Seeds of two carob ecotypes cultivated in Morocco (southern (coastal) ecotype from Essaouira region and northern (in-land) ecotype from Beni-Mellal region) were obtained from the National Institute for Agricultural Research (Marrakesh, Morocco). The southern ecotype (SE), from Essaouira region, grows at a low altitude (not exceeding 200 m), in a warm and semi-arid climate with 340 mm of annual rainfall, and average temperatures ranging from 14.6 °C to 20 °C [86]. The northern ecotype (NE), from Beni-Mallal, grows at a high altitude (1700 m) characterized by a semi-humid climate, with annual precipitation between 700 and 900 mm, and average temperatures ranging from 14 to 15.2 °C. This region is characterized by the availability of surface and groundwater resources [87]. Seeds were scarified in 36 N sulfuric acid for 30 min, rinsed, and submerged in sterile distilled water for 24 h. They were then transferred into Petri dishes on humid filter paper and incubated at 28 °C in the dark for five days. The uniform-looking seedlings were transplanted (one per pot) into 2.8-kg plastic pots filled (4/5) with sterilized soil. The characteristics of the used soil were pH 8.2, 0.9% organic matter, 1.16 g∙kg^−1^ available phosphorus, 12.36 g∙kg^−1^ calcium, and 3.28 g∙kg^−1^ available potassium. At transplanting, half of the plants (30 pots) were inoculated (60 g) with the native arbuscular mycorrhizal consortium inoculum consisting of spores, hyphae, and infected root fragments. The AMF consortium was obtained from five different regions in Morocco: Taounate, Taza, Errachidia, Zagora, and Essaouira. In each region, three sites were selected for collecting the rhizospheric soil samples. The AMF consortium contains a mixture of 25 species of Glomales (*Claroideoglomus etunicatum*, *G. proliferum*, *Rhizophagus clarus*, *Rhizophagus diaphanum*, *Rhizophagus intraradices*, *Funneliformis mosseae*, *Septoglomus constrictum*, *Funneliformis geosporum*, *Diversispora epigeae*, *Glomus* sp1, *Glomus* sp2, *Glomus* sp3, *Glomus* sp4, *Glomus* sp5, *Acaulospora denticulata*, *A. spinosa*, *Acaulospora* sp1, *Acaulospora* sp2, *Acaulospora* sp3, *Acaulospora* sp4, *Acaulospora kentinensis*, *Entrophospora* sp1, *Gigaspora* sp1, *Gigaspora* sp2, *Gigaspora* sp3, *Scutellospora* sp1). The species are divided into four genera (*Glomus*, *Gigaspora*, *Acaulospora*, *Entrophospora*) [88]. AMF consortium was multiplied by trap culture in pots using *Zea mays* L. as the host plant under controlled greenhouse conditions for three months. The consortium was subjected to the most probable number test [89] to determine its potential infectivity and match the applied doses.

Non-mycorrhizal (NM) treatments received an equal quantity of filtered inoculum in an attempt to restore other soil free-living microorganisms accompanying AMF. The filtrate for each pot was obtained by passing the mycorrhizal inoculum in 20 mL of distilled water through a layer of 15- to 20-μm filter papers (Whatman, GE Healthcare, Buckinghamshire, UK). Plants were well irrigated two days per week for six months (from December to May). The non-mycorrhizal and mycorrhizal plants were split each into three treatments: one-third of plants were kept as controls (C) grown under well-water conditions, one-third were exposed to prolonged drought stress by cessation of irrigation for 15 days (15D), and one-third were re-watered for four days (recovery (R)) after the 15D. Plants were grown in a semi-controlled greenhouse at the Faculty of Sciences Samlalia (FSSM, Marrakesh, Morocco) under natural light (photon flux density ranged from 500 to 750 μmol∙m^−2^∙s^−1^). The temperature was maintained at 23/21 °C light/dark. Relative humidity was set to 70%. The experiment used 10 biological replicates for each treatment, and all plants were placed randomly in the greenhouse.

### 4.2. Plant Growth and Mycorrhizal Colonization Measurement

After each harvest, plant height, root length, and shoot and root dry matters (dried at 75 °C until the weight remained constant) were recorded. Fresh roots were cut into 1-cm fragments, washed, and cleaned in 10% KOH at 90 °C for 2 h. The segments were acidified with 5% lactic acid for 20 min, stained with 0.05% (*w/v*) Trypan blue for 30 min at 90 °C [90], and then microscopically observed for root mycorrhizal colonization. The frequency of fungal structures in the root system (F%) and the intensity of the mycorrhizal colonization (M%) were evaluated in 20 randomly chosen root fragments (1-cm length) per glass slide repeated five times for each sample. Mycorrhizal parameters (F% and M%) were calculated according to McGonigle et al. [91], counted from 150 root fragments as follows:

F (%) = 100 × (infected root segments/total root segments),

I (%) = ((95 × n5) + (70 × n4) + (30 × n3) + (5 × n2) + 1 n)/total root segments,

where n is the number of fragments assigned with the index 0, 1, 2, 3, 4, or 5, with the following infection rates: 100 > n5 > 90; 90 > n4 > 50; 50 > n3 > 10; 10 > n2 > 1; 1 > n1 > 0.

### 4.3. Measurements of Physiological Traits

#### 4.3.1. Leaf Water Potential

Leaf water potential (Ψ_Leaf_) was measured using a pressure chamber (SKPD 1400, Skye Instruments, Powys, UK) at predawn (06:00–08:00 a.m.). Measurements were taken on mature fully expanded leaves from the upper part of the stem of each of the five plants per treatment. Cutting leaf water potential was measured over the same days and immediately after gas exchange measurements.

#### 4.3.2. Relative Water Content

Leaf relative water content (RWC) was measured according to the method described by Barrs and Weatherley [92]. Leaf discs (1 cm^2^) for each treatment were cut out from the most expanded leaf (third leaf of the apex), weighed to obtain leaf fresh weight (FW), and immediately hydrated by floating on de-ionized water in a closed petri dish to full turgidity for 24 h under laboratory room light and maintaining them at 4 °C. After hydration, the samples were taken out of the water and were blotted to remove surface moisture and immediately weighed to obtain fully turgid weight (TW). Samples were then oven-dried at 70 °C until the weight was constant to determine the dry weight (DW). The RWC was calculated using the obtained weights for each sample using the following formula:

FWC (%) = (FW − DW)/(TW − DW) × 100,

where FW is the fresh weight, TW is the turgid weight, and DW is the dry weight.

### 4.4. Stomatal Conductance

Stomatal conductance (g_s_) was measured using a portable steady-state diffusion porometer (Leaf Porometer LP1989, Decagon Device, Inc., Washington, DC, USA). Ten measurements per treatment were made on the abaxial side per plant between 9:30 and 11:00 a.m. on sunny days.

### 4.5. Chlorophyll Fluorescence

Chlorophyll fluorescence (F_v_/F_m_) was conducted on the third youngest fully expanded sun-lit leaves using a hand-held fluorometer (Opti-sciences OSI 30p, Hudson, NY, USA). Before measurements, leaves were dark-acclimated for 30 min using leaf clips. The measured F_v_/F_m_ corresponded to the quantum yields (F_v_/F_m_ = (F_m_ − F_0_)/F_m_), where F_m_ and F_0_ are the maximum and initial quantum yields of dark-adapted leaves, respectively.

### 4.6. Mineral Nutrient Content

P, K, Na, and Ca ions in leaves (mg/g DW) were determined by finely powdered plant samples dried in an oven at 80 °C for 48 h and incinerated at 500 °C for 5 h in a furnace. The incinerated material was digested in 2 M HCl. The P concentration was quantified using the Olsen method (Olsen and Dean [93]). The K, Na, and Ca concentrations were measured by flame spectrophotometer (AFP100) as described by Wolf [94].

### 4.7. Analytical Procedures

#### 4.7.1. Total Soluble Sugar and Protein Content

Fully expanded leaves of plants cultured in the presence or absence of AMF under normal, drought, and recovery conditions were harvested at the end of the light period, snap-frozen, and ground to a fine powder in liquid nitrogen with a pestle and mortar. The total soluble sugar (TSS) content was measured in extracts of fresh tissue (0.1 g) homogenized with 4 mL of ethanol (80%). The supernatant was collected and mixed with 0.25 mL of phenol and 1.25 mL of concentrated sulfuric acid. After 10 min, the TSS content was determined by measuring the absorbance at 485 nm according to Dubois et al. [95].

The quantity of total soluble protein was measured by the protein dye-binding method of Bradford [96] using bovine serum albumin (BSA) as a standard.

#### 4.7.2. Determination of the Contents of Hydrogen Peroxide and Malondialdehyde

Hydrogen peroxide concentration was measured spectrophotometrically according to Velikova et al. [97]. Briefly, 0.1 g of frozen fresh leaves were ground with liquid nitrogen to a fine powder and mixed with 5 mL 10% (*w/v*) trichloroacetic acid (TCA) in an ice bath. The obtained mixture was centrifuged for 10 min at 10,000× *g* at 4 °C. The supernatant (0.5 mL) was then recovered, and 0.5 mL of potassium phosphate buffer (10 mM, pH 7) and 1 mL of iodic potassium (1 M) were added. The solution was incubated in the dark for 1 h at room temperature. The absorbance was read at 390 nm and plotted against a standard H_2_O_2_ curve.

Malondialdehyde (MDA) content was determined by homogenizing leaves (0.1 g) in 10 mL of 0.1% (*w/v*) TCA and centrifuged at 18,000× *g* for 10 min as described by Quan et al. [98]. Two milliliters of supernatant were collected, and 2 mL of TCA (20%) containing 0.5% of thiobarbituric acid (TBA) was added. The mixture was then incubated in a water bath at 100 °C for 30 min and immediately cooled in an ice bath to stop the reaction, and then centrifuged at 10,000× *g* for 10 min. The absorbance was read at 450, 532, and 600 nm, and the MDA content was calculated as follows: [MDA] = 6.45 (A_532_ − A_600_) − 0.56 A_450_.

### 4.8. Statistical Analysis

In both carob ecotypes together, the agro-physiological and biochemical variables were subjected to a three-factor ANOVA (factorial 2 × 2 × 3) to partition the variance into the main effects: two ecotypes (NE and SE), inoculation or not with AMF, and the water regime (control, 15D, and R). Significant differences among factors were calculated at 5%. When interaction between factors was significant according to the ANOVA analysis, post hoc comparisons with the Tukey’s test were used to find differences between groups. Each measurement corresponded to the mean of at least five replicates, and mean values and standard deviations were calculated.

## 5. Concluding Remarks and Future Perspectives

In this study, we demonstrated a beneficial effect of AMF at the physiological and cellular levels on improving carob growth, reducing drought damage, and enabling rapid recovery once the stress is removed. In summary, our results demonstrate that the adaptations of mycorrhizal carob to maintain physiological functions could be due, at least in part, to improved water relations, mediating photosynthesis parameters and ion homeostasis, and reducing H_2_O_2_ and MDA content under low plant water status. At the cellular level, AMF-mediated drought tolerance allowed biochemical protection measures to be activated in carob ecotypes in response to drought and recovery to avoid the negative consequences of stress-induced ROS and to withstand water deficit. These abilities to endure drought stress are of great importance for ensuring sustainable crop production under intermittent drought events. In order to corroborate the obtained drought and recovery data illustrating the positive effect of AMF under semi-controlled conditions, the future perspective of this work is to determine the important roles of AMF in drought and ecological stability in large-scale field studies and under more complex environmental conditions.

## Figures and Tables

**Table 1:** Mycorrhizal root colonization frequency (F%) and intensity (I%), shoot height (SH), root length (RL), shoot (SDW) and root (RDW) dry weights of non-mycorrhizal (NM) and arbuscular mycorrhizal (AM) carob ecotypes (northern and southern (NE and SE)) grown under control (C), 15 days of drought (15D), and recovery (R) conditions. AMF—arbuscular mycorrhizal fungi.

		Water Regime	F (%)	I (%)	SH (cm)	RL (cm)	SDW (g)	RDW (g)
NE	NM	C	0.0 ± 0.0 c	0.0 ± 0.0 b	6.7 ± 0.39 ef	15.5 ± 0.59 d	0.77 ± 0.09 cd	0.56 ± 0.08 f
		15D	0.0 ± 0.0 c	0.0 ± 0.0 b	7.3 ± 0.20 bcd	16.9 ± 0.98 c	0.96 ± 0.16 bcd	0.66 ± 0.06 ef
		R	0.0 ± 0.0 c	0.0 ± 0.0 b	7.4 ± 0.21 cd	16.8 ± 0.51 bc	1.09 ± 0.23 abc	0.67 ± 0.10 ef
	AM	C	62 ± 3 ab	32.4 ± 3.1 a	7.3 ± 0.29 cd	18.0 ± 0.74 bc	0.97 ± 0.16 bcd	0.81 ± 0.12 bcde
		15D	65 ± 5 ab	33.9 ± 1.6 a	8.2 ± 0.26 a	19.5 ± 0.49 a	1.40 ± 0.25 ab	0.91 ± 0.11 abc
		R	67 ± 3 a	33.6 ± 3.3 a	8.3 ± 0.11 a	19.6 ± 0.29 a	1.43 ± 0.23 a	0.94 ± 0.16 abc
SE	NM	C	0.0 ± 0.0 c	0.0 ± 0.0 b	6.6 ± 0.15 f	18.8 ± 0.63 ab	0.68 ± 0.15 d	0.63 ± 0.09 ef
		15D	0.0 ± 0.0 c	0.0 ± 0.0 b	6.9 ± 0.27 def	19.7 ± 0.64 a	0.98 ± 0.18 bcd	0.72 ± 0.08 cdef
		R	0.0 ± 0.0 c	0.0 ± 0.0 b	7.0 ± 0.27 de	19.8 ± 0.5 a	1.02 ± 0.21 bcd	0.74 ± 0.13 cdef
	AM	C	62 ± 3 b	34.0 ± 4.3 a	7.5 ± 0.13 bcd	18.7 ± 0.65 ab	0.88 ± 0.13 cd	0.69 ± 0.07 def
		15D	63 ± 6 ab	34.9 ± 8.5 a	8.1 ± 0.48 a	19.9 ± 0.41 a	1.28 ± 0.19 ab	0.96 ± 0.10 ab
		R	72 ± 6 a	38.3 ± 6.1 a	8.1 ± 0.45 a	20.0 ± 0.27 a	1.31 ± 0.10 ab	0.98 ± 0.10 a
Significance								
Water status (A)			NS	NS	*	*	*	*
Ecotype (B)			NS	NS	NS	***	NS	NS
AMF (C)			***	***	***	***	***	***
A*B			NS	NS	NS	NS	NS	NS
A*C			NS	NS	NS	***	NS	NS
B*C			NS	NS	NS	***	NS	NS

Values with the same letter within each column indicate no significant difference among treatments (*p* < 0.05) by the Tukey test. NS—not significant; * *p* < 0.05; ** *p* < 0.01; *** *p* < 0.001. Data represent the means ± standard error (SE) (*n* = 5).

**Table 2:** Nutrient concentrations in leaves of non-mycorrhizal (NM) and arbuscular mycorrhizal (AM) carob ecotypes (NE and SE) grown under control (C), 15 days of drought (15D), and recovery (R) conditions.

		Water Regime	P (mg∙g^−1^ DW)	K (mg∙g^−1^ DW)	Na (mg∙g^−1^ DW)	Ca (mg∙g^−1^ DW)
NE	NM	C	0.31 ± 0.01 d	3.55 ± 0.05 e	3.85 ± 0.18 de	14.64 ± 0.49 fg
		15D	0.32 ± 0.01 c	4.41 ± 0.29 d	4.29 ± 0.17 d	15.57 ± 0.87 f
		R	0.32 ± 0.01 c	4.43 ± 0.39 d	4.33 ± 0.46 cd	16.23 ± 0.18 ef
	AM	C	0.59 ± 0.01 b	6.21 ± 0.15 c	6.05 ± 0.09 b	17.82 ± 1.18 cd
		15D	0.64 ± 0.02 a	8.69 ± 0.38 ab	6.78 ± 0.27 a	19.20 ± 1.06 bc
		R	0.65 ± 0.01 a	8.99 ± 0.13 a	6.87 ± 0.29 a	20.27 ± 0.24 b
SE	NM	C	0.31 ± 0.01 d	3.47 ± 0.18 e	3.50 ± 0.06 e	14.91 ± 0.10 fg
		15D	0.33 ± 0.01 c	4.47 ± 0.22 d	3.89 ± 0.19 de	16.20 ± 0.20 e
		R	0.34 ± 0.01 c	4.54 ± 0.09 d	3.51 ± 0.50 e	16.49 ± 0.29 e
	AM	C	0.59 ± 0.01 b	6.01 ± 0.18 c	5.70 ± 0.26 c	18.40 ± 1.06 c
		15D	0.64 ± 0.01 a	8.34 ± 0.12 bc	6.33 ± 0.37 b	21.20 ± 0.60 a
		R	0.66 ± 0.01 a	8.41 ± 0.23 b	6.33 ± 0.13 b	21.73 ± 0.15 a
Significance						
Water status (A)			NS	*	*	NS
Ecotype (B)			NS	NS	**	*
AMF (C)			***	***	***	***
A × B			NS	NS	NS	NS
A × C			NS	NS	NS	NS
B × C			NS	NS	NS	NS

Values with the same letters within each column indicate no significant difference among treatments (*p* < 0.05) by the Tukey test. NS—not significant; * *p* < 0.05; ** *p* < 0.01; *** *p* < 0.001. Data represent the means ± SE (*n* = 5).
